# Perceptions of primary care staff on a regional data quality intervention in Australian general practice: a qualitative study

**DOI:** 10.1186/s12875-016-0445-8

**Published:** 2016-04-26

**Authors:** Abhijeet Ghosh, Sandra McCarthy, Elizabeth Halcomb

**Affiliations:** COORDINARE - South Eastern NSW PHN, North Wollongong, NSW Australia; Centre for Health Initiatives, University of Wollongong, Wollongong, NSW Australia; School of Nursing, Faculty of Science, Medicine & Health, University of Wollongong, Wollongong, NSW Australia

**Keywords:** Clinical information management, Clinical software, Health data accuracy, Surveillance, Barriers, Clinical data quality, General practice, Primary care data

## Abstract

**Background:**

Technological advances in clinical data capturing and storage systems have led to recent attempts at disease surveillance and region specific population health planning through regularly collected primary care administrative clinical data. However the accuracy and comprehensiveness of primary care health records remain questionable.

**Methods:**

We aimed to explore the perceptions and experiences of general practice staff in maintaining accurate patient health data within clinical software used in primary care settings of regional NSW. Focus groups were conducted with general practitioners, practice nurses and practice administrative staff from 17 practices in the Illawarra-Shoalhaven region of the state of New South Wales (NSW) in Australia that had participated in the Sentinel Practices Data Sourcing (SPDS) project - a general practice based chronic disease surveillance and data quality improvement study. A total of 25 respondents that included 12 general practitioners (GPs) and 13 practice staff participated in the 6 focus groups. Focus groups were audio-recorded and transcribed verbatim. Thematic analysis of the data was undertaken.

**Results:**

Five key themes emerged from the data. Firstly, the theme of *resourcing data management* raised issues of time constraints, the lack of a dedicated data management role and the importance of multidisciplinary involvement, including a data champion. The *need for incentives* was identified as being important to motivate ongoing commitment to maintaining data quality. However, *quality of software packages,* including coding issues and software limitations and *information technology skills* were seen as key barriers. The final theme provided insight into the *lessons learnt from the project* and the increased awareness of the importance of data quality amongst practice staff.

**Conclusion:**

The move towards electronic methods of maintaining general practice patient records offers significant potential benefits in terms of both patient care and monitoring of health status and health needs within the community. However, this study has reinforced the importance of human factors in the maintenance of such datasets. To achieve optimal benefits of electronic health and medical records for patient care and for population health planning purposes, it is extremely essential to address the barriers that clinicians and other staff face in maintaining complete and correct primary care patient electronic health and medical information.

## Background

Rising rates of chronic diseases together with an ageing population pose a serious public health challenge for policy makers and health planners in Australia and internationally [1]. National policies and state-wide approaches to preventive health strategies have been developed [2, 3], but priority-setting for improved population health necessitates region specific local data on disease and health risk prevalence. Monitoring the health status of regional and health administrative area populations is a key function of the planning departments of local health districts and primary healthcare organizations in Australia [4–6]. Accurate disease and health indicator estimates greatly improve understanding of the current chronic disease burden and their ramifications on local health planning, resource allocation and service activity prioritization. However, to achieve accuracy of these estimates requires a constant effort to explore new and innovative methods of data procurement and information collation.

A study conducted in the regional catchment of Illawarra Shoalhaven in the state of NSW, Australia has demonstrated the feasibility of extracting data from general practice clinical software and using the analysis of this routinely collected patient data for local population health surveillance and planning for chronic disease [7, 8]. An integral component of this project involved training general practice staff and clinicians in undertaking data cleansing activities on their respective clinical databases which covered activities that predominantly included: −

- finding all identifiable free-text non-coded past medical history items, and either linking them to appropriate coded items or replacing them with the correct coded items;
- marking patients that had not visited the practice in the last 2 years from the date of collection, as 'inactive' patients
- attempting to have complete demographic records for all patients and not have missing information from patient electronic records such as gender, age, address details and ethnicity
- attempting to have complete health records for all patients and not have key health and clinical information missing from patient electronic records such as height, weight, smoking status, blood pressure etc.

Apart from leading to complete patient health records for population health analysis, these data cleansing activities were aimed to assist clinicians with identifying at-risk patients and inform targeted care and interventions. These were thereby estimated to improve overall patient outcomes and the quality of health service delivery.

Whilst the resultant information from the data collected after the data cleansing phase of the study were shown to provide a potentially reliable and relatively valid estimate of chronic disease prevalence amongst primary care consumers; the study identified an opportunity to improve the accuracy and comprehensiveness of patient electronic health records. Common data entry errors such as missing demographic and/or health information and incorrect/mismatched entries within the collated dataset were still reported as the biggest issues [7–9]. The study and its empirical outputs specifically recommended exploring ways to enhance data accuracy and data quality for furthermore accurate chronic disease and risk factor prevalence estimation.

While theoretically there could be several barriers and issues around data accuracy and completeness of patient electronic health records, it is essential to note that the general practice clinician’s and staff’s awareness of these barriers and the work flows that allow or prevent them to avert these constraints remains the essential piece of the puzzle. Therefore it is extremely important to explore the perceptions of general practice staff on this subject and evaluate their experiences of dealing with data quality issues in line with regional contextual factors.

## Methods

In this paper we aimed to explore the perceptions and experiences of general practice staff during the implementation of an intervention to improve the quality of electronic patient health records in primary care databases aimed at attaining comprehensive population health information on chronic disease and lifestyle risk factors. The other outcomes of this intervention, in terms of disease and risk factor prevalence are reported elsewhere [7–9].

The 17 regional practices that had been involved in the Sentinel Practices Data Sourcing (SPDS) project [7] were selected for this study. The SPDS project is a general practice based chronic disease surveillance and data quality improvement study being conducted in the Illawarra Shoalhaven region of NSW since 2012–13. These practices were purposively selected as they all had undertaken a focused and intensive phase of “data cleansing and enhancement of data accuracy” [7] as part of the SPDS project and so had appropriate experience of undertaking data cleansing and its implications on their patient management and day-to-day practice activities. All clinical (general practitioners and practice nurses) and non-clinical (practice managers and other practice administrative staff) practice staff of these 17 practices were sent an invitation to participate in this study via email.

A convenience sample of 25 respondents that included 12 GPs and 13 practice staff participated in a total of 6 focus groups. Qualitative inquiry using semi-structured focus groups and follow-up individual interviews were conducted. The focus groups were based on the geographic spread of the participating respondents and conducted over a two month period. Each focus group was audio-recorded and then transcribed verbatim. All participants were invited to review their individual transcripts during practice feedback sessions and before finalization of the analyses. No further commentary was received from the participants. Responses were coded using a method of thematic analysis by two researchers separately (one used the manual method and the other used QSR NVivo version 9) [10]. Thereafter the results were discussed with the other member of the research team and inter-researcher agreements on themes and content consensus were reached. Generic texts such as “XXXXX” have been used in the reporting of findings to avoid revealing the identity of participants.

The study was performed with the approval of the Human Research Ethics Committee (Health and Medical) of the University of Wollongong (HE13/433). Written informed consent was obtained from individual participants.

## Results

Five key themes emerged from the data (Table 1).

**Table 1:** Overview of key themes and sub-themes

1. Resourcing data management	
a. Perceived value of data	
b. Time constraints	
c. Lack of dedicated role	
d. Need for multidisciplinary involvement	
e. Importance of a data champion	
2. Need for incentives	
3. The quality of software packages	
a. Coding issues	
b. Software limitations	
4. Information technology skills	
a. Impact of staff age	
b. Turnover of staff	
5. Lessons learnt	
a. Reflection on practice	
b. Ongoing nature of data collection	

### Resourcing data management

Many participants spoke of the importance of quality data to both their practice and patient care. *“Data integrity is very important for the patient’s best care”, “this is a very important thing for our whole community”.* Additionally, participants recognized the role of general practice in participating in data cleansing activities and projects such as the SPDS project that aim to contribute to major health planning decisions and potentially inform policy reform.

*“You tell the community, that this practice is involved in education and research, that it is a thoughtful, scientific community oriented practice, not looking after just the needs of the patient that attend but looking after the wider community attributing to the cutting edge of science”.*

This response recognizes that for real region wide changes to occur the improved procedures will have to be made at the general practice level.

However, despite this recognition of the importance of the task of data management, this did not translate into adequate resourcing of the data management processes. Time was identified as a key barrier for staff; *“it’s another time constraint, it makes things a little bit more time consuming”.* Additionally, data management was seen as an ‘add on’ task rather than a specific role. Therefore, whoever was involved in data management was taking on this responsibility on top of their other workload, causing conflicts in terms of priorities.

*“everybody has their specific role and pretty much their day is filled with that role so to add an additional role onto that would actually mean taking of the admin side of things, taking a person away from that area thereby creating extra work for rest of the girls”*

*“I’m spending a good portion of my day doing a data cleanse, that’s taking me away from other work that I should be doing”*

That created significant challenges for participants who recognized that, *“until you get on top of (this work) it is definitely a full time role”.*

Interestingly, only one out of the 17 practices nominated a GP to be trained in the data cleansing processes, while all the rest of the practices nominated either administration staff or general practice nurses to undertake the training. Whilst many of the data management tasks are administrative, participants without a clinical background identified that they lacked the clinical expertise necessary to undertake all required tasks and often had to delegate and/or consult clinical colleagues.

*“I was part of the team that did the data cleansing, there were a lot of topics that were out of my league with not having a clinical background so I delegate that job to one of our nurses or a group of our nurses when they got time.”*

*“because the receptionists aren’t clinically trained the bits that I could clearly see I fixed but I wasn’t game to change anything else because it then affected things long term.”*

This highlights the need for a multidisciplinary approach to data cleansing and overall data management, including administration, nursing and medical staff. *“You can’t just have one person participating even if it’s a smaller practice, you need all hands on deck”.*

Whilst this multidisciplinary approach was required, there were clear gains when a 'champion' took ownership of the leadership around the project.

*“XXXXX had the other nurses do it …. she is very good at keeping up the protocols and will tell the nurses what to do”*

*“I hadn’t really put it down to the data cleansing project but I think that with XXXXX taking it on, I think there has been an improvement.”*

### Need for incentives

Several participants spoke of the potential for incentives to drive greater engagement in data management. *“Look as human beings we are all driven by incentives be it money or whatever else would be out there so definitely incentives drives all amounts of work”*. In particular the lure of additional funding and upcoming accreditation were seen as major incentives to prompt engagement in data management.

*“there is a lot of income available from general practice management plans again you need to be able to identify people with specific chronic diseases and that’s the way to find them with a clean database that you can search, so it’s not the barriers it’s the lack of incentives”*

*“I suppose if there is a remuneration and a reimbursement for it then I think you will probably see that it’s worth doing and people will do it otherwise people will just carry on doing what they are doing”*

*“If accreditation involved data sampling then I think that this process would get up very very quickly if it was an accreditation standard”*

### The quality of software packages

Two key systemic issues that impacted on participants’ data cleansing and information management experiences arose from the software (EMR software products), namely coding issues and software limitations. In terms of coding, it was perceived that in current medical vocabulary systems in many cases it was difficult to accurately code the patients’ diagnosis. This led to a sense of frustration and encouraged the use of free-text to provide what was perceived to be a clearer and more accurate description of the presenting problem.

*“you look at every permutation and combination to get a code combination of that specific diagnosis and that’s what you want in a patient’s past medical history and the only way you can get it is to free-text it…..”*

*“you start typing it in and it doesn’t come up and it’s because of this infinite diagnoses and it doesn’t come up and you try and think of maybe one or two other things to put in it might come up as that and it doesn’t and you go oh I will just free-text it.”*

Some participants identified that modifications to the coding process to allow both coding and free-text could reduce the impact of this issue on data quality;

*“you have a section where you can have the coded diagnosis but then a note section where you can free-text that additional information, and that additional information goes into the past medical history, so that when you print out a summary … it gets demonstrated on the past medical history”*

The other issue was around the limitations of the software that impacted on both the participants’ confidence in the systems and quality of data.

*“I can run the exact same report 5 min apart and some patients might be left off one report of included in another report so even though it’s the exact same report”*

*“one functional problem in that you can enter a diagnosis, it can indicate that it’s in the database and if you single click it goes back in again as another diagnosis unless you double click and it appears in the box”*

*“it’s too clunky and it doesn’t always work and it’s not the best as of now so again a software issue”*

What was not clear was the contribution of lack of education or skill of the individual participant in terms of them being able to use the range of functions within the software packages.

### Information technology skills

The need for sound IT skills was a key factor impacting on participants. Whilst younger staff were recognized as more likely to have developed computer skills, older staff were identified to frequently have learning needs in this area.

*“we’ve got generally young doctors ….. so they’re right up on all the new things you know”*

*“the younger generation of doctors coming through are more computer savvy and so they’re using the software as it’s meant to be used”*

*“Dr. XXXXX (GP) is one of the oldest in the practice and … his computer skills aren’t great … it adds a lot of time to the consultation”*

Younger staff were also recognized as often being more proactive in using technology in the practice and seeing the potential uses into the future. One older GP commented that younger GPs *“bring with them perceptions and skills and technology that are more visionary certainly than mine”.* In contrast some older GPs were seen as barriers to implementing technology, *“the barrier is just…. Trying to get our older doctor’s to yeah…change”.*

Major barriers to the skill development and training of practice staff were identified as being related to the transient nature of staff such as GP registrars, the turnover of other practice staff and the part-time workforce.

*“in this practice, we’ve had various doctors coming through and I know that there were some who weren’t as good as others”*

*“if they’ve sort of gone from one practice to another or might be here for a short period of time… it’s.. a little bit difficult …to try and enforce them to put things in the way that you want”*

*“we are a teaching practice, so we have registrars, we have GPs that come from the hospital for rotation, so every ten weeks we’ve got a new doctor onboard”*

### Lessons learnt from the study

Participants articulated a number of key lessons learnt from their involvement in the SPDS project. Firstly, they recognized that the project facilitated a level of reflection on their current practice that would not have occurred otherwise.

*“I guess we haven’t stood back and looked at what we’re doing and yeah I guess it’s a bit surprising to see there were a lot of still hand written diagnosis that were old that nobody had changed”*

*“I think that anytime we can step back from data and have a look at it and it can improve the way we interpret it, I think it’s useful.”*

This reflection was seen positively as it assisted in identifying areas for improvement.

Secondly, participants gained an appreciation of the fact that data cleansing and quality assurance is an ongoing task that requires regular commitment to maintain.

*“you can ask the GPs not to free-text but they continue to do it …. Then that corrupts the data so even if you were on top of it, even if you had a completely clean database it’s going to require continued cleansing because of that free-texting that’s being introduced everyday”*

The ongoing nature of the work required to maintain data quality raised issues around how this would be resourced;

*“the doctors probably hopefully are starting to put things in the right places from now on, but as far as going through and cleaning stuff, sitting down for half a day and saying’ I’m going to clean data’, I don’t think that’s going to happen because you know um financially it’s just not viable for them to do that”*

However, involvement in this project had broadly created a heightened awareness and enthusiasm for developing strategies to support data quality activities within the practice.

## Discussion

Our study has highlighted the complexity of maintaining accurate data in Australian general practice settings. Whilst this qualitative study was undertaken in a single geographical region as part of a larger study looking to improve data quality, the themes that emerged describe issues that broadly affect all general practices. What was clear from our study was that a one-sized approach is unlikely to fit the needs of the variety of general practices either within our geographical area or indeed across Australia given the diversity of practice models, individual clinician preferences and variable expertise and enthusiasm. However, some crucial factors were able to be more broadly applied. The engagement of a team of multidisciplinary practice staff in a process of critical reflection upon their current practices and potential barriers and facilitators is important in reaching a mutual understanding of agreed work practices. Additionally, sufficient resources need to be dedicated to the activity of data cleansing and overall data management. There needs to be a clear provision for the workload of data management within either an individual or group of individuals’ roles within the practice and needs to be recognized and remunerated appropriately. Furthermore, by identifying a single enthusiastic 'champion' within the practice who has overall responsibility of data management, means that there is one designated person with the necessary eagerness and skills to maintain data quality who could then lead, engage and encourage everyone else at the practice to also get involved [11, 12].

Many participants in this study spoke of the need for incentives, particularly monetary, to encourage engagement in data cleansing. It was not acknowledged by participants that under Medicare Australia’s e-health Practice Incentives Program (e-PIP recently revised to the Digital Health Incentive under the My Health Record roll-out), monetary incentives are provided for activities that encourage continuing improvements and quality care which include undertaking appropriate clinical coding [13]. However, such incentives would impact practices differently depending on their size and business model. Although monetary incentives may be a motivating factor to maintain data quality, it may also bias data entry and lead to an overestimation of disease conditions or health indicators that attract a higher incentive [14].

In addition to the need to address barriers to general practice staff engaging with data management, this study also highlighted the need to ensure that software systems and coding structures meet the needs of primary care. The validity of the medical terminology and coding systems currently used in primary care clinical software are not sufficiently comprehensive and need further benchmarking and standardization [15]. Additionally there are issues around the interoperability between different terminologies or coding schema that lead to further barriers to data sharing and its meaningful use for planning purposes [16]. Furthermore, the medical vocabularies and schema that are included in all general practice software packages do not automatically update when new conditions are included in classifications of diagnostic criteria [17]. While software vendors could update their medical directories more regularly, this would invariably result in longer lists of drop down menus which has been reported to exacerbate the complexity of using the tool as it becomes difficult for practitioners to search and/or scroll through [12, 18]. This paper opens up the debate and discourse around data management in general practice that has been otherwise rather silent in recent years.

As has been reported in the literature, some participants in this study reported a preference for entering data using free-text rather than selecting from the pre-coded options. Free-text entries are not picked up by any auditing tool/software leading to under-estimation of the true disease and/or risk factor prevalence figures and wrong inferential information for planning purposes [9]. Additionally, the use of free-text can significantly impact on the generation of hazard alerts (patient recalls and/or reminders for practitioners) within the clinical software [19]. While the data cleansing phase of the SPDS study focused heavily on avoidance of any free-text entered into medical or clinical notes by GPs and practice staff through dedicated training and ongoing advocacy of strict free-text avoidance; some level of free-text data entry still continued to occur and remains in practice within primary care settings in Australia. Opinions from participants in this study indicated that sometimes the inflexibility of the currently used primary care medical software in assisting practitioners to code diagnosis in special circumstances such as entering multiple diagnosis in one go or adding supplementary details to the diagnosis being coded leads to clinicians using free-text entries. Free-text entering of data was also seen to be convenient for some clinicians. Software functionality and usability needs to be further researched and developed to make coding more convenient to practitioners than free-texting and have the ability to cater to all circumstances that clinicians face in general practice while eliminating the need for user-developed strategies and workarounds [20].

Another key finding of this study was the impact of the information technology skills of individual staff and the consistent staff turnover upon maintaining data quality. The impact of training needs in relation to information technology of both GP and nurses has been previously highlighted in the literature [19, 21]. Like our study, Chan et al. [22] identified that older primary care clinicians lacked the confidence and skills in using IT in clinical practice compared to their younger colleagues. Future work needs to evaluate various models of providing relevant and effective training that meets the needs of primary care clinicians.

## Conclusion

Practice staff perceive a wide variety of factors to be barriers in maintaining a clean and accurate clinical database. Training on software use and coding will need to be tailored to different sized practices. Findings from this study has the potential to inform further research into the identification of more specific, staff-perceived issues and concerns associated with existing systems and procedures in place for clinical data cleansing, as well as investigating the viability and efficacy of proposed solutions and actions. To be a valid source of data for population level surveillance of chronic diseases, improvements in quality and accuracy of data entered into general practice clinical systems are extremely essential.

### Ethics (and consent to participate)

The study was performed with the approval of the Human Research Ethics Committee (Health and Medical) of the University of Wollongong (HE13/433). Written informed consent to partake in focus groups and interviews and to publish de-identified information in journal publications was obtained from every individual participant.

## Availability of supporting data

All audio tapes and typed transcripts are stored in secure, confidential, password protected storage in the Planning and Performance department of COORDINARE – South Eastern NSW PHN. Completely de-identified participant transcripts could be made available to interested persons/organisations on request to the corresponding author at aghosh@coordinare.org.au.

## Abbreviations

EMR: Electronic Medical Record
GP: General Practitioner
NSW: New South Wales
PIP: Practice Incentives Program
SPDS: Sentinel Practices Data Sourcing
